# Role of Non Receptor Tyrosine Kinases in Hematological Malignances and its Targeting by Natural Products

**DOI:** 10.1186/s12943-018-0788-y

**Published:** 2018-02-19

**Authors:** Kodappully S. Siveen, Kirti S. Prabhu, Iman W. Achkar, Shilpa Kuttikrishnan, Sunitha Shyam, Abdul Q. Khan, Maysaloun Merhi, Said Dermime, Shahab Uddin

**Affiliations:** 1 Translational Research Institute, Academic Health System, Hamad Medical Corporation, PO Box 3050, Doha, State of Qatar; 2 Medical Research Center, Hamad Medical Corporation, Doha, State of Qatar; 3 Translational Cancer Research Facility, National Center for Cancer Care and Research, Hamad Medical Corporation, Doha, State of Qatar

**Keywords:** Non receptor tyrosine kinases, hematological malignancy, signaling pathways, natural products

## Abstract

Tyrosine kinases belong to a family of enzymes that mediate the movement of the phosphate group to tyrosine residues of target protein, thus transmitting signals from the cell surface to cytoplasmic proteins and the nucleus to regulate physiological processes. Non-receptor tyrosine kinases (NRTK) are a sub-group of tyrosine kinases, which can relay intracellular signals originating from extracellular receptor. NRTKs can regulate a huge array of cellular functions such as cell survival, division/propagation and adhesion, gene expression, immune response, etc. NRTKs exhibit considerable variability in their structural make up, having a shared kinase domain and commonly possessing many other domains such as SH2, SH3 which are protein-protein interacting domains. Recent studies show that NRTKs are mutated in several hematological malignancies, including lymphomas, leukemias and myelomas, leading to aberrant activation. It can be due to point mutations which are intragenic changes or by fusion of genes leading to chromosome translocation. Mutations that lead to constitutive kinase activity result in the formation of oncogenes, such as Abl, Fes, Src, etc. Therefore, specific kinase inhibitors have been sought after to target mutated kinases. A number of compounds have since been discovered, which have shown to inhibit the activity of NRTKs, which are remarkably well tolerated. This review covers the role of various NRTKs in the development of hematological cancers, including their deregulation, genetic alterations, aberrant activation and associated mutations. In addition, it also looks at the recent advances in the development of novel natural compounds that can target NRTKs and perhaps in combination with other forms of therapy can show great promise for the treatment of hematological malignancies.

## Background

Tyrosine Kinases (TKs) are a group of around 90 enzymes responsible for catalyzing the transfer of ATP phosphate group to the target protein’s tyrosine residues [1]. This substrate phosphorylation is a mechanism in which activating signals are transmitted from the cell surface to cytoplasmic proteins and the nucleus [2]. In response to external and internal stimuli, TKs have a major role in cell proliferation, survival, differentiation, and metabolism [3, 4]. Recent advancements have identified TKs role in the pathophysiology of cancer, including hematological malignancies [2, 5, 6]. Constitutive or unregulated activity and oncogenic activation in cancer cells is a common pathologic feature and can be blocked by selective TK inhibitors [4–8]. This is therefore considered to be a promising approach for targeted therapeutic development.

The two main classes of kinase are TKs and serine–threonine kinases (STKs) [9]. TKs are further subclassified into receptor and non-receptor proteins. Receptor tyrosine kinases (RTKs) include Platelet-derived growth factor receptors (PDGFR), Fibroblast growth factor receptor (FGFR), Epidermal growth factor receptor (EGFR), and Insulin receptor (IR). RTKs transduce extracellular signals to the cytoplasm and contain a domain that is extracellular ligand-binding, another domain that is intracellular catalytic and responsible for TK activity and regulation, as well as a disulphide bond containing transmembrane domain that connects both the ligand-binding and catalytic regions [9]. RTKs have been shown to be associated with cell division, migration and survival functions, e.g. via the phosphorylation of RAS, initiating RAF-MEK-ERK phosphorylation, consequently resulting in altered gene expression [10].

Non-receptor TKs (NRTKs) are intracellular cytoplasmic proteins that relay intracellular signals [9, 11] and can either be bound to the cell membrane or are nuclear-specific [9]. NRTKs display a broad role in cell signaling. This includes the regulation of gene expression e.g. via IL-6 mediated phosphorylation of the membrane-bound TK (Janus kinase) activating the signal transducer and activator of transcription (STAT) [12]. In addition, inhibition of cell growth e.g. via the stimulation of nuclear TKs (such as Abl) resulting in transcription factor Rb activation [13]. NRTK, such as focal adhesion kinase (FAK), can also regulate cell adhesion and proliferation [14] and are important components of signal transduction pathways, including Fyn [15] and Acks [16]. Furthermore, Acks play a vital role in cell growth via the induction of Janus kinase (JAK) and SRC machinery [17]. Tec family kinases are also associated with intracellular signaling mechanisms [18], as well as SYK which are involved in executing immune response between cell receptors and intracellular signaling [19–21]. Moreover, NRTKs exhibit considerable variability in their structural make up, due to a kinase domain and possession of some protein-protein interacting domains (e.g. SH2, SH3, and PH domain) [4, 22] and additional signaling. Although RTK are activated by ligand-binding, activation of NRTK involves a much more complex mode of action, incorporating heterologous protein-protein interaction, enabling transphosphorylation [23].

STKs however, similar to TKs, can be membrane-bound and nuclear. Additionally, TKs can be transmembrane receptors whereas STKs can also be cytoplasmic [9]. STKs are responsible for the phosphorylation of diverse groups of target substrates, consisting of transcription factors, regulators of the cell cycle, and cytoplasmic and nuclear effector molecules [24]. Certain growth factors, cytokines and physical or chemical induced stress collectively and/or independently act as specific triggers which regulate STK activity [25]. For instance, cytoplasmic STKs (e.g. JNK/MAPK signaling pathway) can be activated by extracellular stimuli resulting in phosphorylated JNK to be translocated to the nucleus stimulating apoptosis through JUN transcription factor [10, 26].

Research now shows that NRTKs, or members of their signaling pathways, show mutations in many forms of hematological malignant cells, which may be in fact dependent on aberrant kinase signaling for their prolonged viability and overall survival. Mutations that lead to constitutive kinase activity however have been found to result in the formation of oncogenes, including ABL, FES, Src, etc. which have been associated in the development of hematopoiesis and their function [2]. Although numerous NRTK oncogenes display differences in their structure, functionality, and subcellular localization, many exploit the same molecular pathways to enhance proliferation and viability [2].

Oncogenic NRTK mutations can be of two types, those due to point mutations, duplications or deletions and insertions, and those involving the development of a fusion gene resulting from a chromosomal rearrangement (e.g. most famously BCR-ABL). NRTK aberrant activation caused by either of these two ways is importance huge cause in the development of many hematological malignancies. Consequently, signal transduction therapy [3] and kinase inhibitors [27] have been sought after to target mutated kinases including those found to be deregulated in various hematological diseases, including lymphomas, leukemias and myelomas. A number of compounds have since been discovered which have been shown to inhibit the activity of NRTKs, which are remarkably well tolerated, considering these compounds typically target a number of kinases, including those both normal and mutant [3].

This review covers the role of various NRTKs in the development of hematological cancers, including their deregulation, genetic alterations, aberrant activation and associated mutations which give rise to such altered expression. This review further aims to showcase how the development of novel natural compounds are able to target kinases and perhaps in combination with other forms of therapy show great promise for the treatment of hematological malignancies. With particular interest in disease states associated with aggressive phenotype and the development of resistance to conventional chemotherapy, we highlight in vivo studies and clinical trials carried out targeting NRTKs with the use of natural products.

## Non receptor tyrosine kinase families

**Fig. 1:**
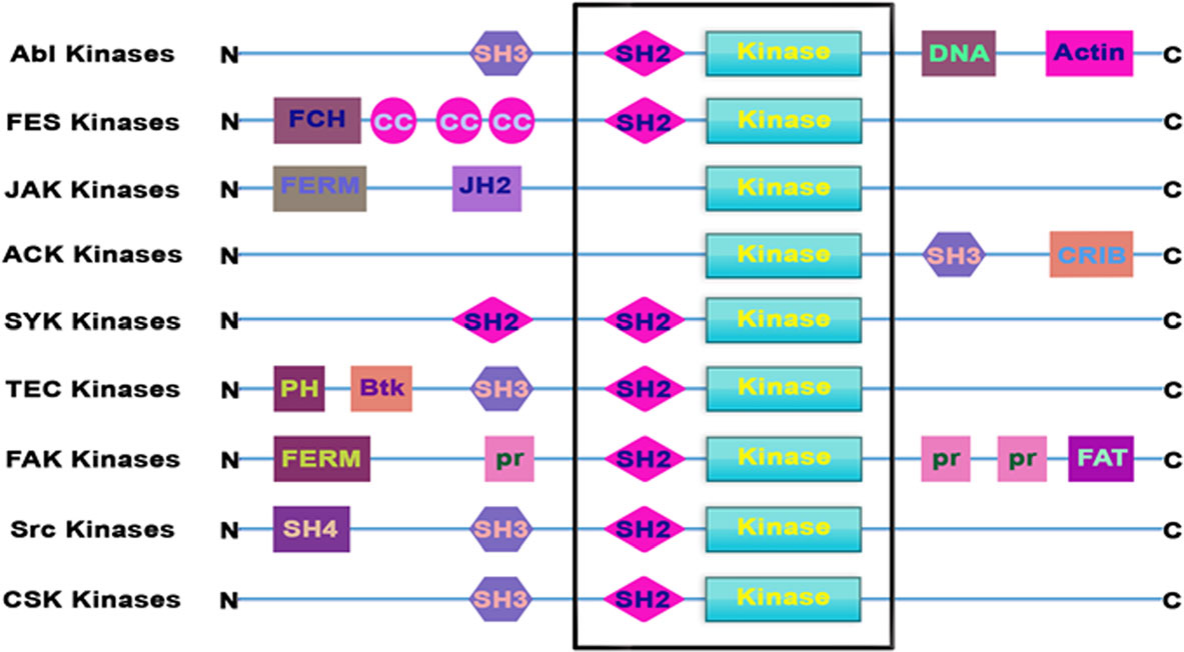
Domain structures of various non receptor tyrosine kinase families. N: Amino terminus, SH3: SRC Homology 3 domain, SH2: SRC Homology 2 domain, Kinase: Catalytic kinase domain (also known as SH1 domain), DNA: DNA binding domain, Actin: Actin binding domain, FCH: Fes/Fer/Cdc-42-Interacting Protein homology domain, CC: Coiled coil motif, FERM: Four-point-one, ezrin, radixin, moesin domain, JH2: Janus homology domain 2 (also known as pseudokinase domain), CRIB: Cdc42/Rac-interactive domain, PH: Pleckstrin homology domain, Btk: Btk-type zinc finger motif, pr: Proline rich region, FAT: Focal-adhesion targeting domain, SH4: SRC Homology 4 domain, C: Carboxy terminus

Non receptor tyrosine kinases are categorized into 9 subfamilies based on sequence similarities, primarily within the kinase domains. These includes Abl, FES, JAK, ACK, SYK, TEC, FAK, Src, and CSK family of kinases (Fig. 1).

### Abl kinases

The Abelson (Abl) kinase family members include Abl1 and Abl2 (Abl-related gene, Arg), which are encoded by both ABL1 and ABL2 genes. This is one of the most conserved branches among the TKs. Human Abl1 and Abl2 proteins are ubiquitously expressed and needed for normal development. Cytoplasmic c-Abl is activated by various growth factors such as PDGF, EGFR, transforming growth factor β and angiotensin subtype 1 receptors [28]. Abl kinases links distinct extracellular stimuli to signaling cascades that regulate cell multiplication and survival, response to DNA damage and stress, dynamics of actin, cell migration, invasion and adhesion [29].

Abl1 and Abl2 kinases have a central SH3-SH2-SH1 (tyrosine kinase) domain unit, with more than 90% sequence similarity among them, and is also shared within the majority of other cytoplasmic kinases. Both have an amino terminal “cap” region and a unique long carboxy terminal tail with various protein-protein interaction sites for proteins such as p53, ATM, etc. This includes a common filamentous actin binding domain (F-BD), Abl1 specific DNA binding domain and globular domain binding with actin upstream of F-BD, and Abl2 specific second F-BD and a domain which is micro tubule binding, upstream of the F-BD. The Abl kinases have a unique cluster of three PXXP motifs, enabling interaction with other SH3 domain containing adaptor proteins such as Abi, Crk, and Nck [30]. Abl1 contains three signal motifs with nuclear localization and in the c-terminal region a nuclear export signal, which regulates its nuclear-cytoplasmic shuttling, while the Abl2 is mainly localized to F-actin rich regions within the cytoplasm and other cellular organelles owing to the lack of any nuclear localization signals [31, 32].

Abl1 was initially thought to be the oncogene vital for the generation of leukemia’s triggered by the Abelson murine leukemia virus. Later identification of the fusion oncoprotein BCR–ABL1 formed by chromosome translocation, t(9;22)(q34.1;q11.2), commonly identified as Philadelphia chromosome (Ph) confirmed the role of Abl family in cancers such as acute myeloid leukemia (AML), chronic myeloid leukemia (CML), and acute lymphoblastic leukemia (ALL), all of which are hematological malignancies. The various malignant Abl fusion gene products encode for constitutively activated Abl kinases that can lead to cellular transformation. In solid tumors, chromosome translocation leading to activation of ABL kinases rarely exists, but is mainly regulated by its over-expression, presence of upstream oncogenic TKs or other chemokine receptors, inactivation of negative regulatory proteins and/or oxidative stress [33, 34].

Numerous intramolecular interactions influencing the SH1 kinase domain can lead to auto inhibition of the catalytic function of Abl kinases. Both SH3 and SH2 domains are involved in the regulation of auto-inhibition. Interactions amid the SH3 domain and the SH2-SH1 linker sequence as well as SH2 domain and the SH1 C-terminal lobe can lead to the formation of SH3-SH2-SH1 clamp structure, which is the auto inhibited conformation. Even a partial disruption of auto-inhibitory constraints results in oncogenic transformation. Inhibition of Abl kinases can also be achieved by interactions with lipids such as phosphatidylinositol 4,5-bisphosphate and myristoylation of amino terminal cap region. The cap region can bind intramolecularly to stabilize the inactive conformation and is required to achieve and maintain inhibition [35]. The abnormal BCR-ABL oncogenic proteins lacks auto-inhibitory cap region and the reintroduction of Abl inhibitory effects upon the reintroduction of cap region conveys the importance of the region in maintaining normal functioning.

Activation of Abl involves extensive domain rearrangements; primarily disruption of SH2 interaction with SH1 c-terminal lobe and in turn binding with the amino terminal lobe of the SH1 domain, leading to allosteric activation that is independent of the ability to bind phosphotyrosine residues. Abl activation can occur through tyrosine phosphorylation in trans by autophosphorylation, SRC family kinases induced phosphorylation and RTKs like PDGFR. Tyrosine phosphorylation of Tyr^412^ in Abl1 / Tyr^439^ in Abl2 positioned inside the activation loop of kinase domain and Tyr^245^ in Abl1 / Tyr^272^ in Abl2 located within the SH2-kinase linker domain is essential to start the kinase activity. Trans-phosphorylation of Abl1 Tyr^89^ located within the binding surface of SH3 domain by Src-family kinases disrupts SH3 domain–based autoinhibition leading to enhanced kinase activity and is obligatory for the full transforming activity of BCR-ABL [36]. Abl1 mediated Tyr^261^ phosphorylation of Abl2 increases protein stability of Abl2 [37], while phosphorylation of Tyr^147^ in the SH3-SH2 connector region of BCR-ABL protein by Src family kinases (Hck, Lyn, and Fyn) modulate BCR-ABL protein conformation and transforming activity [38].

Chronic myelogenous leukemia, a clonal bone marrow stem cell malignancy, is the first human cancer to be correlated with a certain genetic abnormality. CML accounts for 15% - 20% of adult leukemia’s with a frequency of 1–2 cases per 100,000 individuals. It is more common in men and is rarely seen in children. Disruption of the auto-inhibitory intramolecular interactions due chromosome translocation leads to the formation of constitutively active chimeric BCR-ABL1 fusion oncoproteins that drives CML pathogenesis.

Depending on the length of BCR sequence involved during these translocations, 3 distinct BCR-ABL variants may be created, namely p185, p210, and p230. The most common variant in CML is p210 which is observed in hematopoietic cells of CML patients in stable phase, and in ALL and AML [39]. The p230 form is associated with acute leukemia’s, neutrophilic-CML and rare cases of CML. The p185 form is found in about 20–30% of affected adults and about 3–5% of children with B-cell acute lymphocytic leukemia [40]. The loss of cap region inhibition together with the formation of a coiled-coil domain at N terminus of BCR-ABL oncoproteins causes oligomerization and subsequent proximity of numerous kinase domains leading to transphosphorylation of the critical tyrosine residues in the activation loop and other sites contributing to kinase activation [41]. BCR-ABL oncoprotein is the target of the first tyrosine kinase inhibitor (TKI), imatinib mesylate also known as STI571 which is sold under brand name of Gleevec. Majority of the FDA approved kinase inhibitors are currently in clinical use to target BCR-ABL [42]. Imatinib mesylate is an orally available ATP-competitive inhibitor that works by stabilizing the inactive ABL kinase–domain conformation. Nilotinib, Dasatinib, Bosutinib and Ponatinib are second generation TKI used for imatinib mesylate resistant cases.

While BCR-ABL is the most common chromosomal translocation, several other chromosomal abnormalities lead to the expression of various fusion proteins, but there are no activating point mutations identified in the ABL1/ABL2 genes. Various Abl1 fusion proteins involved in hematological malignancies includes BCR-ABL1 (p210), BCR-ABL1 (p185), BCR-ABL1 (p230), NUP214-ABL1, EML1-ABL1, ETV6-ABL1, ZMIZ1-ABL1, RCSD1-ABL1, SFPQ-ABL1, FOXP1-ABL1, SNX2-ABL1, RANBP2-ABL1; while ETV6-ABL2, RCSD1-ABL2, PAG1-ABL2 and ZC3HAV1-ABL2 are originating from Abl2. A large number of signaling pathways are activated by BCR-ABL, but the pathways that are critical for BCR-ABL–dependent transformation includes Gab2, Myc, CrkL and STAT5 [43].

Presence of BCR-ABL oncoprotein is the most frequent genetic abnormality found in adult ALL patients. Nearly 3–5% childhood and 25–40% adult cases of ALL have Philadelphia chromosome, the presence of which confers a worst prognosis and most of these cases present with an aggressive leukemia. First generation tyrosine kinase inhibitor, imatinib mesylate monotherapy can lead to complete remission rates (90%–100%) and combining imatinib mesylate with standard chemotherapy also increases the overall long-term disease-free survival in both adults and children. Imatinib mesylate based induction and consolidation regimens followed by hematopoietic stem cell transplantation significantly improved the outcome Ph^+^ ALL [44].

Approximately 1% of newly diagnosed AML cases show a consistent association with the Ph chromosome [45]. The presentation of cases with CML in myeloid blast crisis and Ph^+^ AML needs stringent criteria to differentiate. Characteristic of Ph^+^ AML includes co-occurrence of typical metaphase chromosome alongside Ph^+^ metaphases during diagnosis, less probability for additional copies of Ph and trisomy 8. Ph^+^ AML patients will have a poor prognosis with standard chemotherapy regimen, and would benefit from combination therapy with imatinib mesylate [46].

### Feline Sarcoma (FES) kinases

FEline Sarcoma (FES) and FEs Related (FER) are members of a separate class of NRTKs called FES kinase family. These kinases are homologous to the viral oncogenes; feline *v-fes* (Feline sarcoma) and avian *v-fps* (Fujinami poultry sarcoma) which are responsible for cancerous transformation. Fes, a 93KDa proto-oncogene, is predominantly present in the myeloid lineage of hematopoietic cells, epithelial, neuronal and vascular endothelial cells, while Fer is ubiquitously expressed. Human c-Fes has been linked to multiple cell surface growth factor and cytokine receptors (ex, interleukin 3 & 4 and GM-CSF receptors) which are involved in cell survival and migration, release of inflammatory mediator and innate immune responses. In addition, it might play a direct part in myeloid differentiation and angiogenesis [47].

Recent findings show that both kinases remain activated in primary AML blasts as well as cell lines. Fes has been reported to have a role in phosphorylation/activation of STAT family of transcription factors, and signaling proteins such as phosphatidylinositol-4,5-bisphosphate 3-kinase, mitogen-activated protein kinases and extracellular signal–regulated kinases [48]. Fes is essential for downstream signaling of the mutated oncogenic KIT receptor. Both Fes and Fer are involved in regulation of vital functions downstream of internal tandem duplication containing FLT3. Fer kinase is necessary for cell cycle progression, while Fes is needed for D816V mutated KIT dependent cell survival.

FES kinases have a unique amino terminal FCH (Fes/Fer/Cdc-42-interacting protein homology) domain, three coiled-coil motifs that facilitate oligomerization, a central SH2 domain for various protein-protein interactions and a kinase domain in the carboxy terminal region. FCH domain together with the first coiled-coil motif is called F-BAR (FCH-Bin–Amphiphysin–Rvs) domain [49]. The biological activity of Fes is tightly regulated, with a tight packing between SH2 and kinase domain to maintain a catalytically repressed state, so that kinase activity is regulated despite the absence of a negative regulatory SH3 domain. Activation of Fes kinase needs active phosphorylation of Tyr^713^ located inside the activation loop. Tyr ^811^ is another critical phosphorylation site for the activation of Fes.

Aberrant activation of Fes is not connected with human cancers. Regardless, studies show that hyper-activation of Fes kinase is critical in maintaining the deregulated proliferation in human lymphoid malignancies elicited by constitutively active forms of mutated surface receptors (internal tandem duplication containing FLT3 and KIT D816V) [50]. Four somatic mutations within the kinase domain of Fes was reported in colorectal cancers, but none of them are gain-of-function mutations [51]. Similarly Fer mutations in small cell lung cancer has been reported [52] Over expression of human c-fps/fes using retroviral vector can transform fibroblasts and other established mouse cells [53] and it requires Ras, Rac, and Cdc42 [47].

### JAK kinases

JAK family of tyrosine kinases consist of four members that includes JAK1, JAK2, JAK3, and Tyk2 [54]. All members of JAKs family contain a similar protein structure; a carboxy terminal kinase domain flanked by a catalytically inactive JH2 (Janus homology domain 2), pseudokinase domain which possesses a kinase-regulatory activity via a SH2 domain. There is also a FERM domain that regulates the binding to the membrane-proximal part of the cytokine receptors [55, 56]. Following binding of the ligand (usually cytokines, such as interferon α/β/γ, interleukins, GPCR ligands and growth factors) to specific receptor, these kinases are activated [57] via tyrosine phosphorylation of the cytoplasmic domains of cytokine receptors [58]. Activated JAKs then subsequently phosphorylate cytoplasmic domain of the receptor [59]. The resulting complex of receptor then recruits and phosphorylates the cytoplasmic STAT family members [60, 61]. STAT family members are major downstream targets of JAK kinases in the pathogenesis oh hematological malignancies [62]. STAT phosphorylation is followed by dimerization and translocation from cytoplasm to the nucleus, where it regulates the manifestation of various target genes [54, 63].

Constitutive activation of JAKs has been reported in many cancers including various hematological malignancies. Deregulated JAK activity arises by numerous means, including aberrant cytokine production via autocrine/paracrine mechanism, activating point mutations within JAKs or any other oncogene upstream of signaling cascade.

Through past several years, a number of JAK mutations that lead to activation of constitutively active or hyperactive JAK activity have been identified [64]. The genetic alteration of JAK family has been reported in all members. It is a well known fact that JAK mutations is linked with development of hematological malignancies [59, 65]. The majority of these alterations are point mutations [59]. JAK2V617F mutation is one of the most studies genetic alteration in JAK family [59]. JAK2V617F mutation is mainly found in primary myelofibrosis or essential thrombocythemia patients. These patients have an incidence of 50% to 60% JAK2V617F mutational freqency and majority (95%) reported polycythemia vera [66]. Another point JAK1 mutation, A634D has been reported in the pseudokinase domain [67]. This mutation has been shown to cause a prominent effect on signaling functions [67]. JAK1 mutation has been found to involve in the development of AML [68] JAK1 mutations are commonly found in T-cell ALL (18%) and with a lesser frequency in B-cell ALL (B-ALL). Constitutive activation of STAT5 has been linked with mutation of JAK1 [65, 69, 70]. JAK1 mutation-mediated activation of STAT5 is also reported in AML patients. JAK3 member of JAK family is found only in hematopoietic lineage. Point mutations leading to aberrant activation of JAK3 have been reported in various leukemia/lymphomas [71]. Juvenile myelomonocytic leukemia (JMML) patients with secondary mutations in *JAK3* have poor prognosis and clinical outcome. In JMML 12% of JAK3 gene has been found to be mutated [72]. Mutation of JAK3 is reported in 15% acute megakaryoblastic leukemia [73]. T-cell lymphoma patients (Extranodal nasal-type natural killer) (21%) were reported to have JAK3 mutations (A573V or V722I) in the pseudokinase domain [74]. These mutations can lead to constitutive JAK3 activation conferring invasive growth and survival advantages. In aggressive T-ALL, JAK3 mutation has been found to be significantly associated [75]. Mutation inTYK2 kinase have been reported in T-ALL (21%) and play a role in promote cell survival via activation of STAT1 as well BCL2 upregulation expression [76].

JAK2 amplification via telomeric segment translocation (9p24) leading to increased JAK2 expression and kinase activity has been described in Hodgkin lymphoma and primary mediastinal B-cell lymphoma [77–79].

### ACK kinases

Acks also known as Activated Cdc42 kinases (Acks) are the important components of signal transduction pathways which comes under the category of non-receptor tyrosine kinases. There are seven different types of Acks viz., Ack1/Tnk2, Ack2, DACK, TNK1, ARK1, DPR2 and Kos1 [16]. Most of the members of the Acks are evolutionary conserved and consists of both N-terminal and C-terminal domains such as a SH3 domain and a kinase domain with key difference in the protein’s c-terminal region [16, 80]. Presence of C terminal kinase domain followed by a SH3 domain along with (CRIB) makes them unique NTRKs [16, 80].

Ack1 (ACK, TNK2, or activated Cdc42 kinase) is one of the most widely studied and first well known members of the Acks. Ack1, a ubiquitous 140KDa protein located on the chromosome 3q, was first cloned in hippocampus of the human brain that binds to active form of CdC42 i.e., in its GTP bound form [80, 81]. Presence of multiple structural domains (N-terminal; SAM domain, tyrosine kinase catalytic domain, SH3 domain, CRIB domain, and C terminal; proline rich domain, ubiquitin associated domain) makes ACK1 distinct from other NRTKs and also provides strong force for its functional diverseness [16, 82].

ACK1 play vital role in cell survival, migration, cell growth and proliferation via acting as an integral cytosolic signal transducer for the array of receptor tyrosine kinases (MERTK, EGFR, PDGFR, IR etc.) to different intracellular effectors which includes both cytosolic as well as nuclear [81]. Furthermore, Ack1 is also an important epigenetic regulator with negative regulatory effect on tumor suppressors [81–86].

Considerable number of reports has revealed crucial role of ACK in the carcinogenesis of various types of neoplasm. Abnormal overexpression, amplification, or mutation of ACK1 has been well documented in many forms of human cancers, including gastric, breast, ovarian, pancratic, colorectal, head and neck squamous cell carcinomas, osteosarcoma, hepatocellular carcinoma, and prostate cancers [81, 85–90]. Recently, Xu et al., revealed that ACK1 promotes development of gastric tumors by p53 ubiquitination degradation via upregulating ecdysoneless homolog, a cell cycle regulator [86] and also reported earlier that ACK regulates the expression of about 147 proteins which are closely associated with cell survival [91].

A number of underlying mechanisms have been documented for ACK1 mediated cancer development. Recently, Maxon et al. reported that mutations in ACK1/TNK2 gene is the main oncogenic cause for AML and chronic myelomonocytic leukemia and that these mutations were sensitive to inhibitors of ACK1 [92]. Furthermore, in the case of chronic neutrophilic leukemia and atypical CML, ACK1 plays critical role in growth by inducing JAK and SRC machinery [17]. In acute leukemia patients harboring NRAS mutation, ACK1 along with other survival proteins have been identified as important therapeutic targets [93]. Diverse crucial role of ACK1 implicated in carcinogenesis including the stimulatory effect on the array of signaling molecules related to cancer development such as AKT, AR, and also by down regulation of tumor suppressors entails its therapeutic importance and prompts the community to look for potential inhibitors.

TNK1 (thirty-eight-negative kinase 1), another important member of ACK family NRTKs of size about 72 KDa, was first reported in blood stem cells of human umbilical cord and murine embryonic cells [16, 94]. Available literature reveals that TNK1 has both tumor suppressing and oncogenic potential as it can mitigate the growth of tumor cells by dowenregulating Ras-Raf1-MAPK pathway [95], induce apoptosis through NF-κB inhibition [96], activate cellular transformation and growth of neoplastic cells [97, 98]. TNK1 sorted out as an important kinase with oncogenic potential implicated in hematological carcinogenesis such as in AML and Hodgkin’s Lymphoma which suggests that targeted intervention of TNK1 may open new platform for therapy.

### SYK kinases

Spleen tyrosine kinase (SYK) is one of the important members of soluble non-receptor protein kinases of syk family and was first cloned in procrine spleen cells with reported highest expression cells of hematopoietic origin [99, 100]. It is a 72 kDa protein encoded by SYK gene located on chromosome 9q22 and consists of highly conserved two SH2 domains with N-terminal and one tyrosine kinase domain at C-terminal and is highest homologous to ZAP-70 [19, 20, 100–102]. SYK is activated by C-type lectins and integrins, and the downstream signaling cascade includes VAV family members, phospholipase Cγ isoforms, the regulatory subunits of phosphoinositide 3-kinases and the SH2 domain-containing leukocyte protein family members (SLP76 and SLP65) [20].

SYK as cytosolic NRTK have crucial role in immune response between cell receptors and intracellular signaling machinery via phosphorylating cytosolic domain of the immunoreceptor tyrosine-based activation motifs (ITAMs) that result in the conformational changes and further activation of SYK which then transduces signal to other downstream target/effector proteins [19–21]. Various findings documented the critical role of SYK in many forms of hematological malignancies by virtue of its stimulatory effect on various survival pathways/signaling molecules [103–105]. SYK has also been found to have tumor suppressive effect in the cancers of non immune cells [106]. Considering important role of SYK in the development of malignancies, progress can be made in the development of effective anticancer molecules.

### TEC kinases

Tec family kinases, the second largest subfamily of the NRTKs, consist of five members, including BTK (Bruton’s tyrosine kinase), ITK/EMT/TSK (interleukin 2-inducible T-cell kinase), RLK/TXK (tyrosine-protein kinase), BMX/ETK (bone marrow tyrosine kinase on chromosome) and Tec (tyrosine kinase expressed in hepatocellular carcinoma) [107]. One of the main features of Tec is the presence of an amino terminal pleckstrin homology (PH) and Btk-type zinc finger (BTK) motif followed by a SH3 and SH2 domains and a carboxy terminal kinase domain in their protein structure. As PH domain can bind phosphoinositides, Tec family kinases are assumed to act as the connection between phosphotyrosine-mediated and phospholipid-mediated signaling pathways. Tec kinases are associated with cellular signaling pathways of cytokine receptors, RTKs, lymphocyte surface antigens, G-protein-coupled receptors and integrins [18]. Tec are abundantly expressed in hematopoietic cells and contribute towards their growth and differentiation [18].

Mutations found in the gene BTK, essential for B-lymphocyte development, differentiation, and signaling [108], have been shown to trigger the human B-cell immunodeficiency, X-linked agammaglobulinemia and X chromosome-linked immunodeficiency in mice. This not only proved that BTK activity is required for B-cell development, but supports the presumption that Tec family proteins are crucial for growth and maturation of blood cells [18]. Previously, the majority of indolent B-cell lymphoma patients did not enter complete remission with treatment and inevitably relapsed [109]. Over the past 10 years, innovative immunochemotherapies have increasingly improved disease control rates but not survival. Therefore, the development of novel agents were urgently needed, which targeted dysregulated pathways in hematological malignancies. In addition, recent preclinical data has illustrated that BTK is present in specific tumor subtypes and in other relevant cells contributing to the tumor microenvironment, e.g. dendritic cells, macrophages, myeloid derived suppressor cells and endothelial cells. BTK inhibitors against hematological malignancies [110] have hence been developed, most notably Ibrutinib (PCI-32765), a first in class covalent inhibitor of BTK. Ibrutinib is an orally available small molecule approved for the treatment of patients with some hematological malignancies and It has been proposed that Ibrutinib may also display antitumor activity in solid neoplasms [111]. Ibrutinib is claimed to be a “breakthrough therapy” by the FDA [109] and overall has changed the future outlook of therapy for lymphoma.

ITKs, the predominant and highly expressed Tec kinase in T cells, act as vital signaling mediators in normal as well as malignant T-cells and natural killer cells [112]. Thus, playing an important role in autoimmune inflammatory diseases [113]. ITK is involved in a variety of downstream signaling from T-cell and NK cell surface receptors and RTKs, mainly the T-cell receptor and Fc receptor [114–116]. ITK mediates signaling by activating phospholipase Cγ1, resulting in activation of downstream targets such as, nuclear factor of activated T-cells (NFAT), NFκB, and mitogen-activated protein kinase pathway [117]. ITK inhibitors could hence have therapeutic potential in several autoimmune, inflammatory, and malignant diseases. For example, in a recent study by Zhong et al. [112], using the novel ITK/RLK inhibitor PRN694, ex vivo assays reported inhibitory activity against T-cell prolymphocytic leukemia cells.

TXK expression is mainly detected in some myeloid cell lines and T-cells. Moreover, TXK is expressed in T-cell subsets (e.g. Th1/Th0 cells), and was reported to act as a Th1 cell-specific transcription factor, regulating IFN-γ gene expression via binding to its promoter region, increasing transcriptional activity [118]. An increasing amount of interest has focused on T-cell subsets, which have been characterized, based on their array of cytokine production, e.g. Th1 cells have been found to secrete IL-2, IFN-γ, and lymphotoxin, supporting cell-mediated response [118–122].

BTK, ITK, and TXK have shown selective expression in bone marrow cells [123]; however BMX and TEC have displayed a much broader expression, even extending to normal somatic cells (e.g. cardiac endothelium) [107]. BMX has been reported to be expressed in myeloid lineage hematopoietic cells (e.g. granulocytes and monocytes), endothelial cells, and numerous types of oncologic disorders [107]. Over the past decade, there was significant progress made in this area of research, which has suggested a prominent role of BMX in cell survival, differentiation and motility, and as such, a key player in inflammation and cancer [107, 124].

TEC is expressed in hematopoietic cells like myeloid lineage cells, B and T cells, as well as neutrophils and has been reported to be involved in the stabilization of lymphocytes (B and T), T and B cell receptor signaling, and in the nuclear factor activation of activated T-cells [125]. The overexpression of TEC has been found to be associated with tumorigenesis and liver cancer progression [126]. Inhibiting TEC or degrading the phosphorylation of TEC may therefore have a direct affect on the progression and development of liver cancer. This was supported by an investigation carried out by Chen et al. [127] exploring TEC protein expression in hepatocellular carcinoma and TEC phosphorylation in 200 specimens of cancerous and non-cancerous liver tissue. A more recent study by Vanova et al. [128] with interest in the expression of TEC in hepatocellcular carcinoma, identified TEC as a regulator in controlling pluripotent cell fate in human pluripotent stem cells, acting through the regulation of fibroblast growth factor-2 secretion. Such studies provide further support and evidence of the broad activities and roles of tyrosine kinase preferentially expressed in hepatocellular carcinoma.

### Focal adhesion kinase

FAK family consists of 2 members; the ubiquitously expressed focal adhesion kinase, and the associated adhesion focal tyrosine kinase (Pyk2) that is manifested in the central nervous system and in hematopoietic cells. FAKs play a part in the normalization of cell propagation and adhesion as well as cell to microenvironment communications [14]. FAK over expression has been reported in B-lymphoblastic leukemia and lymphoma cells. Interestingly, FAKs are absent in leukemias/lymphomas of T-cell origin as well as in myeloma [129]. FAK responds to extracellular stimuli, including signals from the extracellular matrix to regulate cell proliferation and migration [130]. Interaction of growth factor with receptor has been found to activate leading to phosphorylation/activation of Src kinase. The activated Src kinase then, via association with several signaling pathways, regulates cell proliferation and survival of cancer cells [131]. FAK expression is found to be significantly higher in MDS patients with CD34^+^ and CD34^+^ CD38^−^ as compared to patents with normal CD34^+^ [131]. FAK overexpression has been linked to the enhancement of leukemic cell migration from the marrow to the circulating compartment, associated with drug resistance [132]. FAK regulate cell migration through regulation of Rho family of proteins, paxillin kinase linker (PKL/Git2)–β-pix complex and phosphatidylinositol 4,5-bisphosphate. FAK overexpression in AML has been found to be associated with poor survival outcome [132, 133].

### Src kinases

The Src family of tyrosine kinases (SFKs) are membrane-associated NRTKs active as key mediators of biological signal transduction pathways. This family includes 11 related kinases: Blk, Fgr, Fyn, Hck, Lck, Lyn, c-Src, c-Yes, Yrk, Frk (also known as Rak) and Srm [134, 135].

SFK members share a highly conserved structure, comprising of a membrane-targeting myristoylated or palmitoylated SH4 domain in the amino terminal region, trailed by SH3, SH2 and a kinase domains, and a short carboxy terminal tail with an auto-inhibitory phosphorylation site [134]. Furthermore, each member of SFKs has a specific domain of 50–70 residues that is consecutive to the SH4 region and divergent among the different family members [136].

Five members of the SKFs (Blk, Fgr, Hck, Lck and Lyn) are expressed predominantly in hematopoietic cells. However, c-Src, c-Yes, Yrk and Fyn, are expressed ubiquitously with high levels in platelets, neurons and some epithelial tissues [134, 137]. Moreover, Srm is present in keratinocytes, and Frk expressed primarily in bladder, breast, brain, colon, and lymphoid cells [135].

SFKs have a major role in a variety of cellular signaling pathways activated through various RTKs (PDGF-R, EGF-R, FGF-R, IGF1-R, CSF-R) [138] and G-protein coupled receptors, regulating cell survival, DNA synthesis and division, actin cytoskeleton rearrangements and motility [137, 139]. Src family member exerts its full catalytic activity upon phosphorylation of a critical residue (Tyr^419^) within the activation loop. Phosphorylation of the auto-inhibitory site Tyr^530^ within the carboxy terminal tail forms a closed auto-inhibited inactive conformation via the association of the SH2, SH3, and kinase domains by intramolecular interactions. Many factors, including specific cellular signals, or transforming mutations, could break these interactions and produce an active open kinase [140]. Several protein tyrosine phosphatases can dephosphorylate Tyr^530^ and thus regulate its kinase activity.

SFKs associate with PDGF-R via an interaction of their SH2 domain with Tyr^579^ of the ligand bound activated receptor. This association will release the auto-inhibitory intra-molecular interface between the SH2 domain and the carboxy terminal tail, subsequently permitting the formation of catalytically active conformation. SFKs in turn modulate RTK activation and are involved in promoting mitogenesis.

SFKs might have a part in cancer development due to their implication in the regulation of cell–cell adhesion. This regulatory pathway involves different molecules such as p120-catenin protein, a substrate of Src [141]. SFK, particularly Src, might also be involved in tumorigenesis by activation of STAT transcription factors which regulate cytokine signaling in hematopoietic cells [142]. Moreover, SFKs like focal adhesion kinase, paxillin and p130CAS have been implicated in monitoring of signaling pathways mediated by integrin. Alterations in integrin activity have been described in several tumor types [143]. Src is also thought to have a role in the progression of CML, AML, CLL, and ALL through activation of STAT pathways and regulation of RAS/RAF/MEK/ERK MAPK and VEGF pathways. Other oncogenic pathways of C-Src include translocation of B-catenin, promotion of ERK and Cbl phosphorylation and increase in anti-apoptotic Bcl2 in cancer cells [144–146].

SFKs also play a role in the development and signaling of T and B cells. Indeed, SFKs, particularly Lck, appear necessary for T cell receptor-based signaling essential for various phases of T-cell development [134, 147]. In addition, Lyn, have a major role in B-cell lineage development and maturation, activation as well as inhibition [148].

A consistent number of studies point the role of SFKs in human tumors since they are often overexpressed and/or constitutively hyper-activated in several cancers [137]. Activation of SFKs could arise either after a mutation of Src allele leading to disrupted negative regulatory network or to binding of SFKs to activating partners such as growth factor receptors (Her2/Neu, PDGF, EGFR, c-kit), adaptor proteins and other NRTKs (focal adhesion kinase, Bcr-Abl) [149]. Various SFKs members have been implicated in the development of hematopoietic malignancies such as leukemia and lymphomas (AML, ALL, CML, Burkitt’s lymphoma, etc.) [150]. However, oncogenic mutations of SFKs are rarely observed in hematological malignancies [151]. Therefore, the progression of leukemia and lymphoma malignancies is mainly associated with the constitutive activation of SFKs and to amplification of anti-apoptotic and oncogenic downstream signaling pathways [149, 150].

In cancer cells, multiple mechanisms are able to disrupt the inactive conformation of SFKs including binding of SH2 to activated receptors such as flt3 (in AML) and to oncogenic protein kinase such as BCR-ABL (in CML and ALL) [152]. Furthermore, in cancer cells, the SFKs inhibitory signaling pathways such as C-terminal Src kinase have shown to be suppressed thus leading to a stabilization of SFK activated conformation [151]. Activation of SFKs promotes multiple downstream signal transduction cascades implicated in apoptosis and oncogenesis (STAT3 and STAT5, MEPK, EGFR, PDGFR, PI3K/AKT and VEGFR) [146, 149, 150, 153].

Moreover, it has been shown that SFKs promote cancer cells resistance to chemotherapy and radiation as well as targeted RTK therapies [154, 155]. Donato et al. have demonstrated that Lyn and Hck, were upregulated in imatinib mesylate resistant cell line and in specimens of advanced CML and ALL from patients who relapsed to imatinib mesylate [149, 156]. Indeed, SFKs members, particularly Hck and Lyn, interact with the oncogenic BCR-ABL fusion protein and promote resistance to imatinib mesylate treatment [157].

Given the importance of SFKs in multiple aspects of tumor development, such as proliferation, migration, resistance to apoptosis, and angiogenesis, these proteins can be considered as attractive targets for future anti-cancer therapeutics. Moreover, inhibition of SFKs in combination with standard anti-cancer therapies has been suggested as a promising treatment strategy with a clinical potential in overcoming resistance to current regimens and preventing metastatic recurrence [154].

The viral encoded Src (v-Src) is constitutively active and highly transforming, where as c-Src over expression does not induce transformation. v-Src transformed cells, but not c-Src over expressing cells, have the ability to form tumors in nude mice [158]. But mutant form of c-Src created by single amino acid changes (Thr to Ile at position 338/Glu to Gly at position 378/Phe to Ile at position 441) or by fragment of c-src (Gly-63, Arg-95, and Thr-96) with a corresponding fragment of v-src (Asp-63, Trp-95, and Ile-96) is oncogenic and induce transformation ([159, 160].

Fyn has been found to be overexpressed in various types of cancers including hematological malignancies [15, 161, 162]. Fyn is involved in development and activation of T cells [15]. Activated Fyn is proven to play a role in the pathogenesis of multiple human carcinomas via influencing cell growth, transformation ability of cells and apoptosis [15]. Fyn has been also found to participate in generation of mitogenic signaling, initiation of cell cycle and cell to cell adhesion [163]. Fyn also play a critical role in aggressiveness of CLL.

Lyn is aberrantly expressed and highly activated in many cancer cells [164, 165]. Association of Lyn kinase with dysregulated signaling pathways in various hematopoietic as well solid tumors implicates that it might be an important target for the treatment of cancer. Dysregulation of Lyn have an important role in progression of CLL via regulation of apoptotic signaling pathway [166]. A number of substrates has been identified in CLL including SYK, PI3K, HS1, procaspase-8, and PP2A [167–169].

### C-terminal Src kinases

C-terminal Src kinases (Csk) and Csk-homologous kinase (Chk) are the two members of this family of NRTKs. Csk is a 50 kDa protein with an amino terminal SH3 domain followed by a SH2 domain and a carboxy terminal kinase domain. A characteristic feature of Csk is the absence of a site in the activation loop for auto-phosphorylation. The active conformation is stabilized by the binding of SH2-kinase and SH2-SH3 linkers to the amino terminal lobe of the kinase domain.

CSKs phosphorylates the auto-inhibitory tyrosine residues in the Src-family kinases’s C-terminal tail which stabilizes SFKs in a closed inactive conformation and thus functions as the major endogenous negative regulators of SFKs. Chk can engage a complementary mechanism to inhibit SFKs by direct binding to SFKs, which is also called as non-catalytic inhibitory mechanism. Several other signaling proteins such as paxillin, P2X3 receptor, c-Jun and Lats can also serves as substrates of Csk, but the physiological relevance of it is not yet known [151, 170].

Csk is ubiquitously expressed in all cells, however, Chk is mainly expressed in the brain, haematopoietic cells, colon tissue and smooth muscle cells [170]. Csk is primarily present in cytosol as it does not have a transmembrane domain or any fatty acyl modifications. As the substrate molecules (SFKs) are attached to the membrane, the mobility of Csk to the membrane by means of numerous scaffolding proteins (caveolin-1, paxillin, Dab2, VE-cadherin, IGF-1R, IR, LIME, and SIT1), is a crucial step in the regulation of Csk activity [151].

They have an important role in the regulation of cell functions like growth, migration, differentiation, and immune response. Recent studies suggest that Csk can have a function as tumor suppressor through the inhibition of SFKs oncogenic activity.

## Targeting NRTKs using Natural products

Past few years have seen tremendous improvement in the area of drug discovery in field of cancer. Although many new entities are available in market for therapeutic treatment but association of adverse events like acute/chronic organ damage, suppression of bone marrow, and potential toxicities like hepatic, renal, gastrointestinal etc., with these drugs limits its use [171–174] continuing quest for search of newer and effective molecule.

Nowadays targeted therapy are gaining high importance due to its ability to directly act on specific molecule and signaling pathway. Tyrosine kinases compete with ATP binding site of the catalytic domain of oncogenic tyrosine kinases and modulate signaling pathway [175]. Thus it becomes very important to target such kinases by using specific drugs that aims directly at kinases.

Inhibitors like IFN-alpha regulates T-cells however, due to patient non-compliance and side effects produced thereby limited its use. Second generation tyrosine kinase inhibitors like dasatinib, nilotinib, bosutinib along with imatinib mesylate have gained tremendous respect as a conventional chemotherapeutic agent for treatment in CML patients. Despite of great achievements in the therapeutic treatments of CML, search for newer effective and potent agent toward resistant mutants such as T315I is continued. Drugs like aurora kinases, ponatinib were effective against resistant mutant but due to cardiac toxicity and maximum tolerated dose for ponatinib being 45 mg their use were limited [5, 175–181]. Natural products are now considered as an alternative for synthetic drugs.

**Fig. 2:**
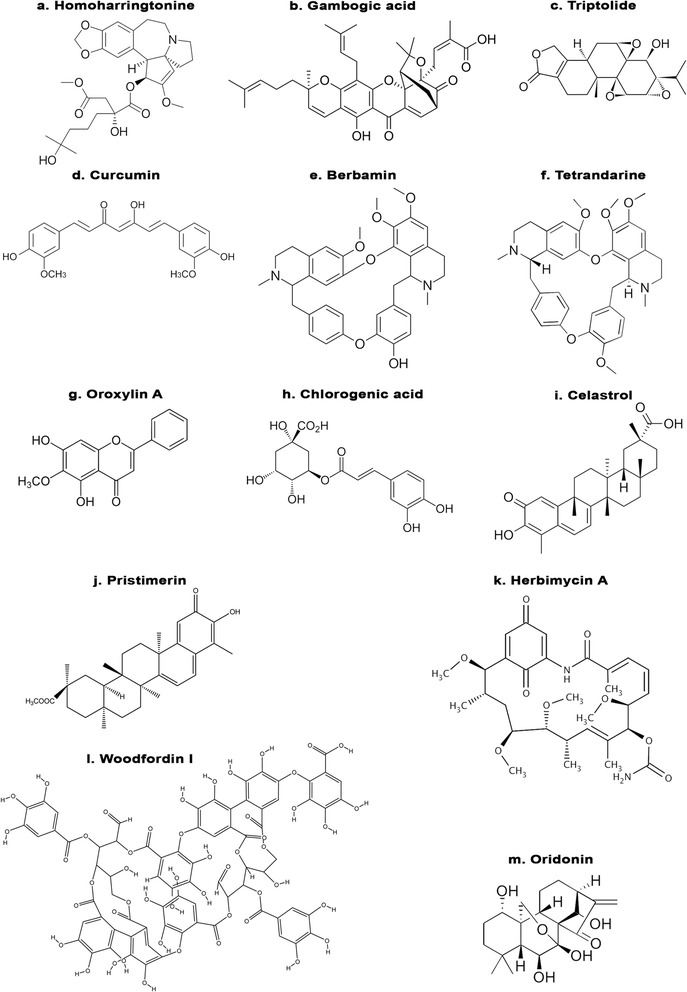
Chemical structures of various natural products targeting non receptor tyrosine kinases

Secondary metabolite/s present in natural products is known to possess diverse biological effects. These natural products are present in numerous sources like plants, micro-organism, fungi etc. Besides being non-toxic in nature they are considered to less expensive. In 2013, US FDA approved 1453 new chemical entities from which 40% comprised of natural products or analogs of natural compounds [182, 183]. Natural products alone or in combination have been able to induce apoptosis as well chemosensitised those cell lines that were resistant to conventional drugs. Below we have discussed compounds (Fig. 2) that hold a high potential to be developed as a lead molecule as a tyrokinase inhibitor. Some of these natural compounds has in vivo (Table 1) data and some has progressed through clinical trials (Table 2).

**Table 1:** In vivo studies of natural products against hematological malignancies

HSS(Haishengsu)	Nude mice	450,900,1800 mg/kg bw, intravenous	↓Bcr-Abl	[213]
Triptolide	Nu/nu BALB/c mice	0.15 mg/kg Bw, subcutaneous	↓Bcr-Abl	[214]
curcumin	Balb/c mice	40 mg/kg bw, intraperitonial	↓c-ABL	[216]
Berbamine(BBM)	Balb/c	60 mg/kg bw, intravenously	↓p-gp protein	[220]
4-Chlorobenzoyl berbamine(BBD9)	NU−/−	15 and 30 mg/kg BW,	↓P-BCR-ABL	[223]
Tetrandrine citrate	Nu−/−	100 mg/kg BW, orally	↓BCR-ABL	[225]
homoharringtonine	BABL/c mice	0.5–1 mg/kg BW, intraperitonial	↓Bcr-Abl	[186]
Oroxylin A	SCID	80MG/KG BW, intravenously	↓STAT3,p-gp	[227]
chlorogenic acid	Nude mice	25-150 mg/kg bw, intraperitonial	↓Bcr-Abl	[228]
Celastrol	Nu/nu BalB/c Mice	1 mg/kg BW, intraperitonial	↓Bcr-Abl	[229]
Pristimerin	nu/nu BALB/c mice	1 mg/kg Bw, intratumoral	↓Bcr-Abl	[230]
Herbimycin A	Balb/c nude mice	15 μg,intraperitonial	↓abl	[232]

**Table 2:** Natural products against hematogical malignancies in clinical trials

NATURAL PRODUCTS	CANCER TYPE	STATUS	STUDY PHASE	Clinical trials.gov number
Homoharringtonine	CML	COMPLETED	PHASE 2	NCT00375219 (a)
Tanespimycin (17-AAG)	AML	COMPLETED	PHASE 1	NCT00098423 (b)

^a^ https://clinicaltrials.gov/ct2/show/record/NCT00375219?term=omacetaxine&cond=LEUKEMIA&draw=1&rank=2

^b^ https://clinicaltrials.gov/ct2/show/NCT00098423?term=17-AAG&cond=Leukemia&draw=1&rank=6

### Omacetaxine mepesuccinate

Omacetaxine mepesuccinate is also known as homoharingtonine (HHT) (Fig. 2a) is a cephalotaxine ester isolated from bark of *Cephalotaxus* species and belongs to class of alkaloidal compounds. Documented reports claim that practioners from Fujian Province of China used the extract of HHT in treatment of cancer. However limited availability of this drug led to development of semisynthetic compounds with better efficacy and safety compared to parent drug. It is only one such natural drug that owes US FDA approval for treatment of CML patients that developed resistant or failed to respond to conventional chemotherapeutic tyrokinase inhibitor. The liver is able to metabolize Omacetaxine mepesuccinate without causing liver toxicity and have biphasic half life of alpha 0.5 ± 0.1 h and beta half life of 9.3 ± 1.4 h [184, 185].

Omacetaxine has been studied using cell lines (BCR-ABL- expressing myeloid and lymphoid) and in in vivo mouse model of CML and B-cell acute lymphoblastic leukemia bearing BCR-ABL or BCR-ABL-T3151 mutation. Both in vitro and animal model results demonstrated reduction in number of leukemic cells in both CML and in mice models. Besides this, omacetaxine also repressed expression of BCR-ABL-T3151 expressing leukemic cells [186, 187]. Similar effects were demonstrated in study comprising of omacetaxine mepesuccinate and nilotinib in combination [188]. However, published data specifies that omacetaxine does not rely on BCR-ABL binding site to exhibit its activity instead it blocks synthesis of protein by competing with amino acid side chain of aminoacyl-tRNAs to bind with A-Site cleft of ribosomal unit. [189, 190].

In the early 1980s omacetaxine mepesuccinate was used in the treatment of CML patients. In clinical trial (phase I/II), six patients who did not respond to conventional chemotherapeutic tyrosine kinase inhibitor imatinib mesylate, responded to omacetaxine mepesuccinate treatment. This evaluable effect was observed in five patients. Complete blood related response was observed in all patients whereas three of them showed genetic response at cellular levels. Efficacy of treatment was measured on basis of decreasing levels of BCR-ABL transcripts [191].

Cytogenetic response along with absence of BCR-ABL mutation was witnessed in two patients harboring BCR-ABL kinase mutation before start of omacetaxine treatment [192]. Efficiency of omacetaxine mepesuccinate was studied in Phase II clinical trial consisting of patients bearing BCR-ABL-T1351 mutation. Median rate of seven cycles of omacetaxine mepesuccinate treatment were received by 62 patients. Out of 62, 48 patients showed signs of complete hematologic response whereas, 14 patients achieved major cytogenetic response. Progression free survival rate was reported to be for 7.7 month [193].

So far couples of phase II clinical studies have verified effect of HHT and omacetaxine mepesuccinate in patients with CML who were in early stage of the disease or in the late chronic phase. In all 212 CML patients received either HHT or omacetaxine mepesuccinate at dose of 2.5 mg/m^2^ for 14 days or 1.25 mg/m^2^ twice a day for 14 days respectively. Complete hematologic response rate was 80% whereas cytogenetic rate was found to be 42% [190, 192–194]. In another clinical study consisting of 252 patients who did not respond or developed resistance to two or more tyrosine kinase inhibitor were treated with omacetaxine mepesuccinate at a dose of 1.25 mg/m^2^ twice a day for 28 days and then less than 7 days/cycle as maintenance dose showed 20% of cytogenetic response [195, 196].

Omacetaxine mepesuccinate was also tried in tandem with other treatment agents and drugs. Efficiency of omacetaxine mepesuccinate was tested in combination with imatinib mesylate at dose of 1.25 mg/m^2^ twice a day for 14 days on 24 chronic phase CML patients in of. Complete blood and genetic response at cellular level of 66 and 55% respectively was achieved [191, 197]. Another study comprising of 225 patients with CML were evaluated for HHT at dose of 2.5 mg/m^2^ and ara-c combination treatment. Complete hematologic response of 81% was achieved upon this treatment [198, 199]. Combination treatment therapy using 2.5 mg/m^2^ of HHT with interferon alpha showed complete blood response at rate of 89% and cytogenetic response rate of 57% in CML patients in early chronic phase [200]. Average complete blood response rate of 94% and cellular genetic response rate of 74% were demonstrated in 90 CML patients receiving either HHT, interferon alpha and ara-C [201].

Omacetaxine mepesuccinate, in addition to being used in CML treatment, has also found its place in treatment of acute and multiple myeloma. Study conducted with continuous administration of HHT (5 mg/m^2^) for 9 days in 16 patients with myelodysplastic syndrome and 12 patients with myelodyplastic syndrome exhibited response rate of 28%. Complete and fractional remission in seven and one patient respectively was observed [202]. Phase II pilot study consisting of HHT at dose of 2.5 mg/m^2^ when given in form of infusion for 7 days followed by maintenance dose failed to show any response in eight patients [203].

Study was conducted in 66 patients with relapsed acute myeloid leukemia or blastic phase CML to evaluate efficacy of HTT. 16% of patients showed sign of complete remission. Two patients that were resistant to cytarbine showed complete remission whereas 11 patients resistant to cytarbine did not respond to treatment with HTT [204].

Clinical trials clearly justify the potential of HHT or its semi synthetic form in treatment of CML and other blood related disorders. However, along with its positive response hematological side effects like granulocytopenia, thrombocytopenia, myelosupression and non haemotological toxicities like diarrhea, fatigue, nausea, head ache chest pain etc. were commonly observed during course of treatment.

### Gambogic acid

Gambogic acid (Fig. 2b) is a phytoconstituent belonging to class of xanthones which was isolated in form of gum resin form *Garcinia hanburryi* (also known as mangosteen). Xanthones, is a class of secondary metabolites isolated from plant, fungi and lichens, exhibits wide spectrum of activities such as anti-cancer, anti inflammatory, anti diabetic etc. [205–208]. Presently, the Chinese FDA has approved gambogic acid and phase II clinical trials are underway [209]. Development of imatinib mesylate resistance due to presence of BCR-ABL T3151 mutation in CML cells has led to search of novel therapeutic agent/s. Shi X group demonstrated the apoptotic effect of gambogic acid on CML cells, mononuclear cells from imatinib mesylate -sensitive or -resistant patients and in xenograft tumor model bearing T315I-BCR-ABL genes or wild-type BCR-ABL. It was observed that gambogic acid successfully induced apoptosis along with inhibition of cell proliferation in cell lines sensitive or resistant (bearing KBM5-T315I mutation as well as in the mononuclear cells from imatinib mesylate resistant patients) to imatinib mesylate. In xenograft model gambogic acid reduced tumor growth in nude mice harboring T315I-BCR-ABL mutation [209]. Structure-activity relationship (SAR) study by Sun et al., [210] showed that the caged 4-oxa-tricyclo[4.3.1.03,7]dec-2-one xanthone scaffold was the key pharmacophoric motif of gambogic acid. Molecular docking studies have shown that gambogic acid and its derivatives can bind to the ATP binding pocket of IKKβ and make several H-bonds with the hinge region of the enzyme, leading to inhibition of IKKβ. But there are no SAR studies with gambogic acid on any of the NRTKs.

### Haishengsu

Haishengsu is a protein molecule with molecular weight of approximately 15KDa obtained from shell fish *Tegillarca granosa*. This compound is expected to help improve clinical results in the case of patients with renal and lung cancer when used alone or as an adjuvant with conventional chemotherapeutic drug/s [211, 212]. I*n* in vivo *study m*ice bearing drug resistant leukemia cell lineK562/ADM(Adriamycin) Haishengsu acted by repressing levels of multi drug resistant gene-1 (mdr1), BCR-ABL and sorcin at dose of 1800 mg/kg which was significant in comparison to group that received no drug (control) and that received adriamycin and haishengsu in combination [213].

### Triptolide

Triptolide (Fig. 2c) is isolated from leaves of *Tripterygium wilfordii* was studied for its effect on KBM5 cell lines (wild-type BCR-ABL, T315I mutant BCR-ABL) and on peripheral blood mono nuclear cells from imatinib mesylate resistant patients. Triptolide induced time and dose dependent apoptosis in KBM5 cells and in peripheral blood mono nuclear cells. Induction of apoptosis was accompanied with decreased expression of BCR-ABL, phosphorylated STAT5, CrkL and Erk1/2. In vivo study, using imatinib mesylate -resistant CML cells in nude mouse xenograft model triptolide inhibited tumor proliferation without encouraging much appreciable changes in bodyweight. Immnuno histochemical analysis supported claim of triptolide in downregulating BCR-ABL [214]. Synthesis and biological activity studies of Triptolide derivatives have shown that the C-14β–OH group, C-9,11-β-epoxide group, C-12,13-α-epoxide group, C-7,8-β-epoxide group, the 5-membered lactone ring and the C-5,6-position are critical for the cytotoxicity and antitumor activities of Triptolide [215]. But there are no specific studies relating to NRTKs.

### Curcumin

Curcumin (Fig. 2d) is an alkaloid isolated from various Curcuma species decreased viability of cells and promoted apoptosis in cells isolated from B6 mice expressing wild-type BCR-ABL (B6p210) and T315I mutant (B6T315I) human leukemic cells. B6p210 cells bearing p210 oncogene was found to be more susceptible to treatment then compared to T315I mutant. Western blot and transcription factor assay revealed occurrence of apoptosis via inhibition of c-abl and NF-kB along with its downstream targets. Beside this upregulation of p53 was also observed in both B6p210 and B6T315I cells. Improvement in survival rate and white blood cells/GFP positive cell counts compared to control was observed in mice with B-ALL [216]. Studies with synthetic derivatives shows the involvement of phenolic OH group in the antioxidant activity [217]; methoxy group in on inflammatory responses and NF-κB signaling [218] in the biological activities of curcumin, while there are no SAR studies relating its function to the NRTKs.

### Berbamine

One of the major component isolated from chinese herbal medicine is *Berberis amurensis*. It is an effective calcium channel blocker [219]. Besides being used as calcium channel blocker it has promising effects against chronic myeloid leukemia, breast cancer, and melanoma [220–222]. Wei et al., demonstrated the ability of berbamine (Fig. 2e) to reverse mdr-1 along with reduced expression of P-glycoprotein both in in vitro and in vivo models [220]. Similar results were observed on use of 4-chlorobenzoyl berbamine (BBD9) an analogue of berbamine in imatinib mesylate resistant cells (K562/IR) in vitro and in vivo. BBD9 lowered expression of p210BCR-ABL, IKKα, nuclear NF-κB p65 along with its downstream target [223, 224].

### Tetrandrine

Tetrandrine (Fig. 2f) belongs to class of bis-benxylisoquinoline alkaloid isolated from *Stephania tetrandra*. Although tetrandrine has gained importance due its ability to inhibit several tumor cells in vitro but poor solubility has limited its use. Xu-Xh et al.*,* 2012 studied effect of salt form of tetrandrine (Tetrandrine citrate) on imatanib resistant K562 cells bearing high expression levels of p210(BCR-ABL). Decrease in expression levels of p210(BCR-ABL), β-catenin and BCR-ABL at mRNA level were observed in imatinib mesylate resistant K562 cells in vitro. Nude mice bearing imatinib mesylate resistant K562 cells showed complete sign of regression with no symptoms of toxicity when orally administered with tetrandrine citrate at dose of 100 mg/kg body weight [225]. SAR study unveiled the role of -OCH_3_ group present in a particular benzene ring of tetrandrine in the regulation of non-voltage-operated Ca^2+^ entry and release of intracellular Ca^2+^ in human promyelocytic leukemia cells [226], while there are no studies relating its structure to NRTKs.

### Oroxylin A

Oroxylin (Fig. 2g) (5,7-dihydroxy-6-methoxyflavone) is an O-methylated flavonoid isolated from herb *Scutellariae baicalensis* when studied in combination with imatinib mesylate resulted in marked depletion of pY^705^-STAT3 along with its downstream targets p- glycoprotein in imatinib mesylate resistant K562 cells. NOD/SCID mice bearing K562 cells demonstrated decrease in tumor volume and weight significantly in oroxylin A (80 mg/kg) and imatinib mesylate (200 mg/kg) combined group compared to control and drug alone group [227].

### Chlorgenic acid

Chlorgenic acid  (Fig. 2h)is isolated from leaves of *Piper betel* promoted cell death by hindering expression of BCR-ABL and c-Abl kinases through activation of p38 and ERK-MAP kinase in cells bearing positive BCR-ABL and in BCR-ABL–positive leukemic cells isolated from CML patients in vitro. Sodium salt of chlorogenic acid due to its better solubility was found to be more sensitive compared to parent compound. Reduction in cancer advancement in nude mice bearing K562 xenograft was observed with salt form of acid [228].

### Celastrol

Celastrol (Fig. 2i) is an active triterpenoid isolated from *Tripterygium wilfordii* which reduces active levels of phospho BCR-ABL and total BCR-ABL in CML cells bearing wild-type BCR-ABL and in T315I mutant (BCR-ABL resistant to imatinib mesylate) cells. In vivo study demonstrated effect of celastrol in reducing size and weight of tumor in imatinib mesylate resistant and imatinib mesylate sensitive cells nude xenograt model. Reduced levels of c-abl and BCR-ABL were further confirmed by immunohistochemical analysis. Combinational therapy using celastrol and 17-AAG (tanespimycin or geldanamycin) for 72 h showed synergistic/co-additive inhibitory effect [229].

### Pristemerin

Pristemerin (Fig. 2j) is a quinonemethide triterpenoid isolated from *Celestraceae* and *Hippocrateaceae* family species promoted cell death by inhibiting growth of CML cells. Pristemerin induced dose dependent decrease in levels of p-BCR-ABL and total BCR-ABL at protein and mRNA levels as detected in imatinib mesylate sensitive (KBM5), imatinib mesylate resistant (KBM5-T3151) cells lines and in K562 cells. Concomitantly phosphorylation of CRKL, STAT5, AKT were also decreased with little or minimal effect on total AKT and STAT5. Significant tumor growth inhibition was observed compared to control in imatinib mesylate resistant BCR-ABL-T3151 xenografts in nude mice. Authors also confirmed that BCR-ABL reticence preceded apoptosis [230].

### Herbimycin A

Antibiotic herbimycin A (Fig. 2k) is isolated from culture filtrate of Streptomyces species MH237-Cf-8, at their non cytotoxic concentration reduced levels of p210^c-abl^ and induced erythroid differentiation in K562 and KU812 cells obtained from Ph + leukemic patients [231]. Similar results were observed in study conducted by same group of researcher wherein treatment with herbimycin A induced differentiation and prolonged survival time of nude mice inoculated with C1 cells expressing high level of protein tyrokinase [232, 233]. Herbimycin A and its synthetic analogue 17-cyclopropylamino-herbimycin A and 4,5-dichloro-herbimycin inactivated various tyrokinase like src, c-abl, BCR-ABL [234].

### Woodfordin I

Woodfordin I (Fig. 2l), is a macrocyclic ellagi tannin dimer isolated from *Wodordia fruticose* and Denbinobin isolated from *Cannabis sativa* reduced expression of p120 c-Abl, p210 BCR-ABL, c-Abl and BCR-ABL in K562 human leukemic cells [235, 236].

### Oridonin

Oridonin (Fig. 2m) is a diterpenoid isolated from *Rabdosia rubescens* inhibited lyn and abl levels along with downstream target Akt/mTOR, Raf/MEK/ERK and STAT5 in Ph + ALL cell line and primary specimens from Ph + ALL patients. Oridonin with imatinib mesylate exerted in tandem effects by overcoming imatinib mesylate problem of upregulating Akt/mTOR and lyn signaling [237].

Substantial evidences focusing on the potential of numerous phytoconstituents in inhibiting carcinogenis using in vitro and in vivo models in diverse cell systems have been published [238, 239]. Phytoconstituents like apigenin, resveratrol etc. that are found in wide range of fruits and vegetables and are gaining prominence owing to its ability to induce apoptosis via loss of mitochondrial membrane potential and caspase activation in K562 sensitive and K562/IMA3 (K562 cell resistant to 3uM imatinib mesylate) cells [240–243]. In addition to this and above mentioned natural compounds like fiestin, hesperidin, virosecurinine, cryptotanshinone, quercetin, genistein vincristine, and many more had competence when used alone or in combination with other tyrosine kinase inhibitor to down regulate BCR/ABL and lyn levels in cells developing resistance to imatinib mesylate and CML patients [244–249].

## Conclusion and Future Perspectives

Non receptor tyrosine kinase are involved in multiple signaling pathways that regulate vital functions such as cell proliferation and differentiation, and plays a role in human neoplasms, inflammatory and autoimmune diseases. Clinical use of highly successful tyrosine kinase inhibitors (such as imatinib Mesylate, Herceptin and Gefitinib) endorse the potential of targeted cancer therapy using specific NRTK inhibitors. Targeted therapy has the advantages of being less toxic than traditional cytotoxic chemotherapy due to specificity for cancer cells. The best example is imatinib mesylate, a maximum limit of dose could not be identified during the phase 1 clinical trials. Unfortunately, even targeted therapy with the FDA approved small molecule NRTK inhibitor, omacetaxin mepesuccinate, have hematological side effects and other drawbacks. Some of these side effects may be due to the inhibition of other related tyrosine kinases present in normal cells and therefore extremely difficult to avoid completely. Nevertheless, additional knowledge of the side effects will make possible to develop better targeted drugs that are able to avoid these limitations.

In the case of general TK inhibitors like imatinib mesylate, development of resistance (due to point mutations or gene amplification) has become the major challenge. Similarly, resistance towards NRTK inhibitors could also develop in patients. A long term strategy to design rapid and efficient biochemical and cell based high throughput assay for screening of novel kinase inhibitors is needed. Implementation of bioinformatics based methodologies (structure based drug design, based on the current knowledge of the three dimensional structures of target kinases, mathematical quantitative modeling of cancer progression and drug response, etc) can hasten the process of screening several natural compounds through the drug discovery process.

Although several natural compounds have proven their efficacy in in vitro and in vivo models as potent tyrokinase inhibitors, in-detail research is still required to establish natural compounds as lead molecules for clinical trial. Till date only one single natural compound, Homoharringtonine, has been able to successfully finish clinical trials and receive an FDA approval. A key obstacle in the development of a specific inhibitor is the variation in efficacy observed in the cell line based experiments and rodent models during drug discovery phase leading to the final efficacy in patients. NRTK inhibitors may well add invaluable contribution to treatments in combination with conventional chemotherapy.

## Abbreviations

ALL: Acute lymphoblastic leukemia
AML: Acute myeloid leukemia
ara-C: Cytarabine
CML: Chronic myeloid leukemia
CRIB: Cdc42/Rac-interactive domain
CSK: C-terminal Src kinase
EGFR: Epidermal growth factor receptor
FAK: Focal adhesion kinase
F-BAR: FCH-Bin–Amphiphysin–Rvs domain
F-BD: Filamentous actin binding domain
FCH: Fes/Fer/Cdc-42-interacting protein homology
HHT: Homoharingtonine
IFN: Interferon
JAK: Janus kinase
JMML: Juvenile myelomonocytic leukemia
NRTK: Non receptor tyrosine kinase
PDGFR: Platelet-derived growth factor receptor
Ph: Philadelphia chromosome
PH: Pleckstrin homology
RTK: Receptor tyrosine kinase
SFK: Src family of tyrosine kinase
STAT: Signal transducer and activator of transcription
STK: Serine-threonine kinase
TK: Tyrosine Kinase
TKI: Tyrosine kinase inhibitor
